# Supplementary material for: An open-source framework for large-scale, flexible evaluation of biomedical text mining systems
Source: J Biomed Discov Collab. 2008 Jan 29;3:1. doi: 10.1186/1747-5333-3-1 (PMC2276192; doi:10.1186/1747-5333-3-1)
Supplement: Additional file 1 — Evaluation platform source code. The additional file contains the software evaluation framework discussed in this paper. The most current release can be downloaded from SourceForge.net [1]. See the accompanying README file for instructions on installation and use. Also included in the distribution is a collection of UIMA wrappers for some commonly used BioNLP tools and annotated corpora. [file 1747-5333-3-1-S1.zip › bionlp_uima_repository-1.0.1/docs/api/edu/uchsc/ccp/util/nlp/mention/Mention.html]

Mention (Center for Computational Pharmacology Util/UIMA API)


|  |  |  |
| --- | --- | --- |
| **Overview** \| **Package** \| **Class** \| **Tree** \| **Deprecated** \| **Index** \| **Help** | |  |
| **PREV CLASS**   **NEXT CLASS** | **FRAMES**    **NO FRAMES**     **All Classes** |  |
| SUMMARY: NESTED \| FIELD \| CONSTR \| METHOD | DETAIL: FIELD \| CONSTR \| METHOD |  |


---


## edu.uchsc.ccp.util.nlp.mention Class Mention

```
java.lang.Object
  edu.uchsc.ccp.util.nlp.mention.Mention
```

**All Implemented Interfaces:**: Comparable

**Direct Known Subclasses:**: ClassMention, ComplexSlotMention, SlotMention

---

``` public class Mention extends Object implements Comparable ```

---

**Constructor Summary**

`Mention()`
  
          **Deprecated.**

`Mention(String mentionName)`


| **Method Summary** | |
| --- | --- |
| `int` | `compareTo(Object o)`             Must be implemented by subclasses of Mention |
| `String` | `getDocumentLevelSingleLineRepresentation()` |
| `String` | `getMentionName()` |
| `String` | `getSingleLineRepresentation()` |
| `void` | `setMentionName(String mentionName)`             **Deprecated.** |

**Methods inherited from class java.lang.Object**

`clone, equals, finalize, getClass, hashCode, notify, notifyAll, toString, wait, wait, wait`

**Constructor Detail**

### Mention

```
@Deprecated
public Mention()
```

:   **Deprecated.**

---


### Mention

```
public Mention(String mentionName)
```


**Method Detail**

### getMentionName

```
public String getMentionName()
```

---


### setMentionName

```
@Deprecated
public void setMentionName(String mentionName)
```

:   **Deprecated.**

---


### getSingleLineRepresentation

```
public String getSingleLineRepresentation()
```

---


### getDocumentLevelSingleLineRepresentation

```
public String getDocumentLevelSingleLineRepresentation()
```

---


### compareTo

```
public int compareTo(Object o)
```

:   Must be implemented by subclasses of Mention

    :   **Specified by:**: `compareTo` in interface `Comparable`
    :   **Parameters:**: `o` - **Returns:**


---


|  |  |  |
| --- | --- | --- |
| **Overview** \| **Package** \| **Class** \| **Tree** \| **Deprecated** \| **Index** \| **Help** | |  |
| **PREV CLASS**   **NEXT CLASS** | **FRAMES**    **NO FRAMES**     **All Classes** |  |
| SUMMARY: NESTED \| FIELD \| CONSTR \| METHOD | DETAIL: FIELD \| CONSTR \| METHOD |  |


---
